# Causation of Phthisis

**Published:** 1868-03

**Authors:** 


*Causation of Phthisis.*—Dr. Budd has astonished the English physicians by a new theory of the origin of this " endemic of the universe," as Dr. Bartlett used to call it. He thinks it a zymotic disease, like typhus or scarlet fever; never originating spontaneously but always transmitted by pre-existing germs These germs are the tuberculous matter itself which is transmitted from patient to patient, and which bears the same relation to the disease as the vesicles do to small-pox. He hopes that the destruction of this matter on its issue from the body will ultimately enable us to " stamp out" consumption entirely.

He thinks there is direct evidence of transmission from one person to another, an opinion long held by the Spanish physicians of the island of Cuba. He also maintains that many countries were free from this disease till it was introduced from Europe. He cites the South Sea Islanders, the North American Indians and the natives of Africa, who are all said to have known nothing of phthisis until civilization brought them its questionable blessings. Even now, he tells us that in Africa phthisis only prevails on the coast where the tribes come in contact with the whites, while in the interior where their intercourse is only rare and occasional, it is still unknown. Dr. Livingtone is cited as authority for the latter statement.

This is certainly a startling theory, and the acknowledged sagacity of Dr. Budd should secure for it at least a patient hearing and a candid investigation.

